# Supplementary figures and images for: Dorsal raphe stimulation relays a reward signal to the ventral tegmental area via GluN2C NMDA receptors
Source: PLoS One. 2023 Nov 6;18(11):e0293564. doi: 10.1371/journal.pone.0293564 (PMC10627466; doi:10.1371/journal.pone.0293564)

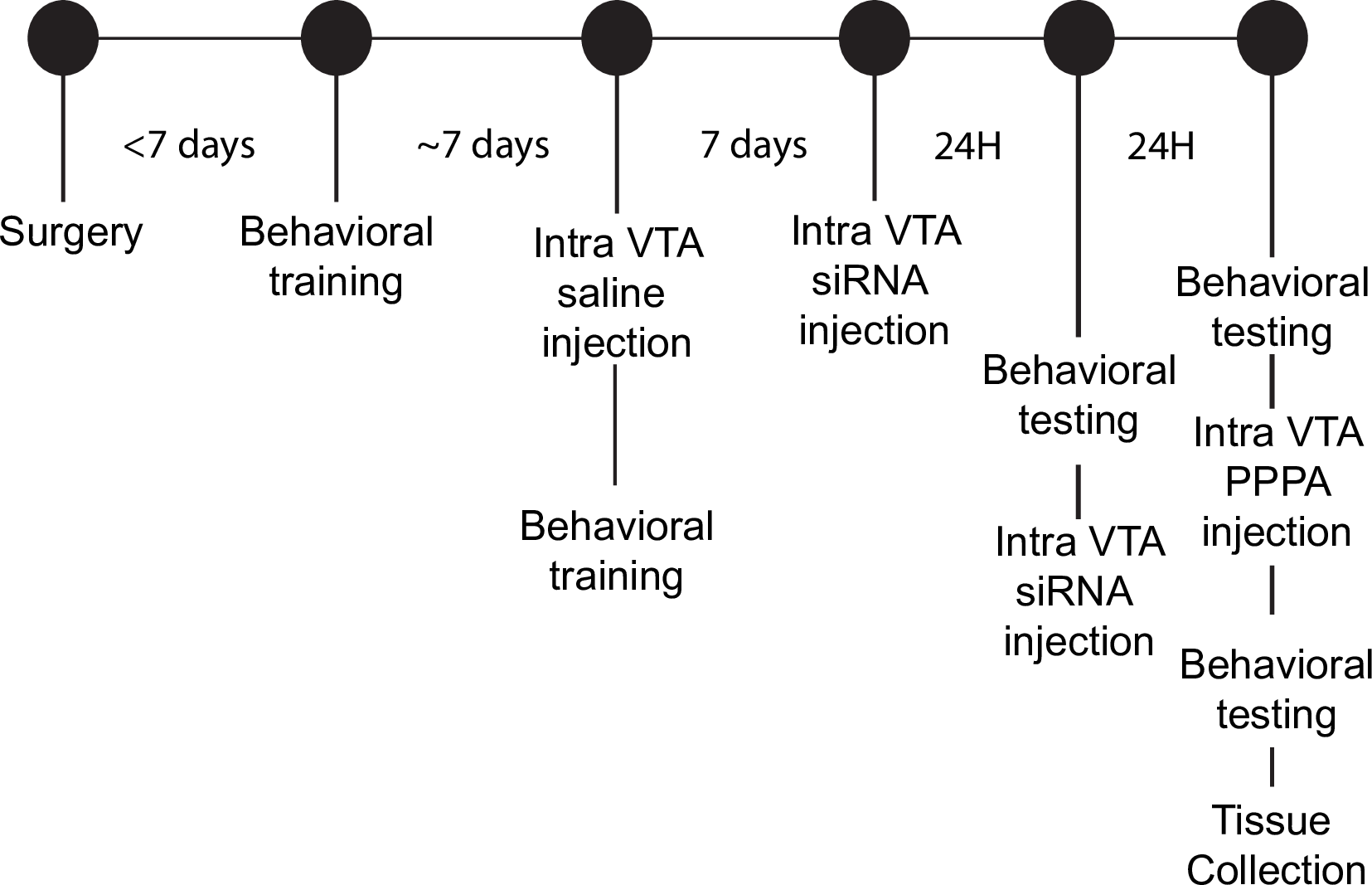

Supplement: S1 Fig — (TIF) [file pone.0293564.s001.tif]

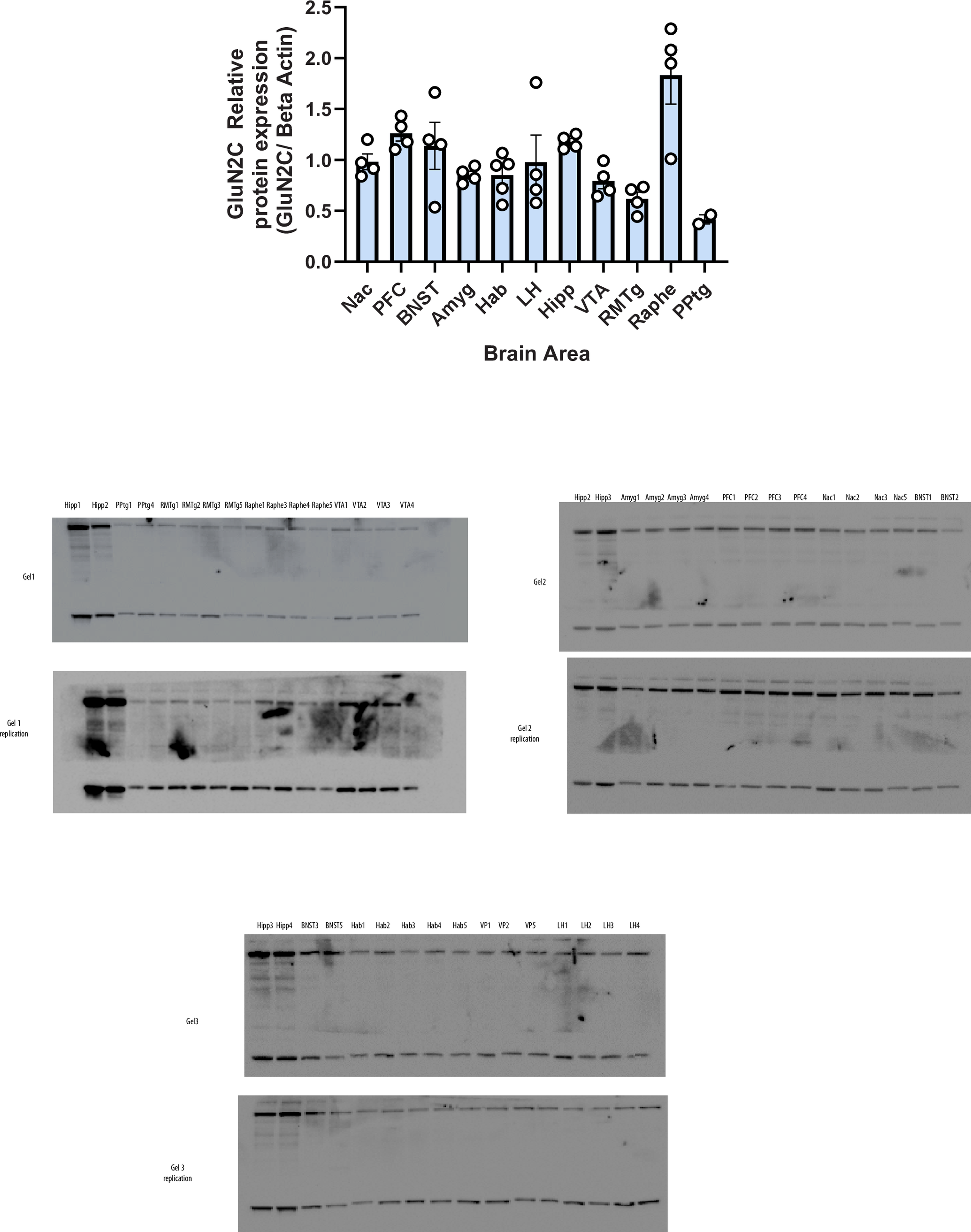

Supplement: S2 Fig — (TIF) [file pone.0293564.s002.tif]

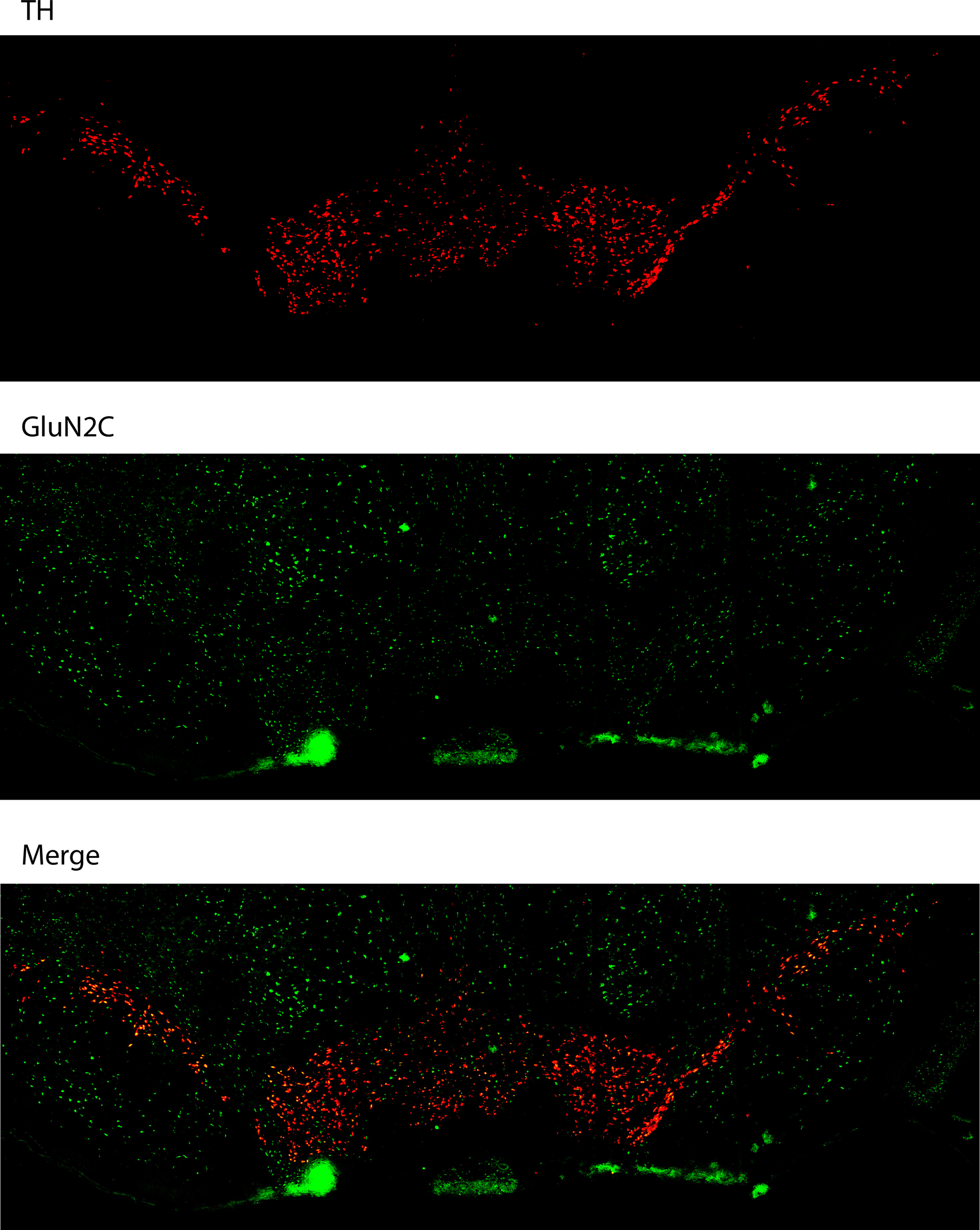

Supplement: S3 Fig — (TIF) [file pone.0293564.s003.tif]
