# Supplementary material for: Dorsal raphe stimulation relays a reward signal to the ventral tegmental area via GluN2C NMDA receptors
Source: PLoS One. 2023 Nov 6;18(11):e0293564. doi: 10.1371/journal.pone.0293564 (PMC10627466; doi:10.1371/journal.pone.0293564)
Supplement: S1 Table — (DOCX) [file pone.0293564.s004.docx]

**Primer sequences for qPCR Grin2 analysis**

|  |  |
| --- | --- |
| \|  \| **Gene Symbol** \|  \| **Fwd** \| **Rev** \| **RefSeq** \| \| **Efficiency %** \| \| \| --- \| --- \| --- \| --- \| --- \| --- \| --- \| --- \| --- \| \|  \|  \|  \|  \|  \| \|  \| \|  \| \| \|  \| Grin2a \|  \| atgatcatggctgacaagga \| cagcataactgtggcttgct \| \| NM_012573.3 \| \| 102 \| \| \|  \| Grin2b \|  \| catggtatctcgcagcaatg \| cagcgctgaatggctctaa \| \| NM_012574.1 \| \| 91 \| \| \|  \| Grin2c \|  \| ggcactcctgcaacttctg \| gttctggcagatccctgaga \| \| NM_012575.3 \| \| 100 \| \| \|  \| Grin2d \|  \| gcccattctcgacttcctg \| aggaaggtggagcccttct \| \| NM_022797.1 \| \| 107 \| \| \|  \| Gapdh \|  \| ccctcaagattgtcagcaatg \| agttgtcatggatgaccttgg \| \| NM_017008.3 \| \| 97 \| \| \|  \| Hprt \|  \| gaccggttctgtcatgtcg \| acctggttcatcatcactaatcac \| \| NM_012583 \| \| 105 \| \| | |
|  | |
